# Diachronic profile of startup companies through social media

**DOI:** 10.1007/s13278-023-01055-2

**Published:** 2023-03-18

**Authors:** Ana Rita Peixoto, Ana de Almeida, Nuno António, Fernando Batista, Ricardo Ribeiro

**Affiliations:** 1 Instituto Universitário de Lisboa (ISCTE-IUL), ISTAR, Lisbon, Portugal; 2 NOVA Information Management School (NOVA IMS), Universidade Nova de Lisboa, Campus de Campolide, Lisbon, Portugal; 3 INESC-ID Lisboa, Lisbon, Portugal; 4 Instituto Universitário de Lisboa (ISCTE-IUL), Lisbon, Portugal; 5 CISUC – Center for Informatics and Systems of the University of Coimbra, Coimbra, Portugal; 6 CITUR, Centro de Investigação, Desenvolvimento e Inovação em Turismo, Faro, Portugal

**Keywords:** Topic modeling, Social media, Startups, Life cycle model, Twitter data

## Abstract

Social media platforms have become powerful tools for startups, helping them find customers and raise funding. In this study, we applied a social media intelligence-based methodology to analyze startups’ content and to understand how their communication strategies may differ during their scaling process. To understand if a startup’s social media content reflects its current business maturation position, we first defined an adequate life cycle model for startups based on funding rounds and product maturity. Using Twitter as the source of information and selecting a sample of known Portuguese IT startups at different phases of their life cycle, we analyzed their Twitter data. After preprocessing the data, using latent Dirichlet allocation, topic modeling techniques enabled the categorization of the data according to the topics arising in the published contents of the startups, making it possible to discover that contents can be grouped into five specific topics: “Fintech and ML,” “IT,” “Business Operations,” “Product/Service R&D,” and “Bank and Funding.” By comparing those profiles against the startup’s life cycle, we were able to understand how contents change over time. This provided a diachronic profile for each company, showing that while certain topics remain prevalent in the startup’s scaling, others depend on a particular phase of the startup’s cycle. Our analysis revealed that startups’ social media content differs along their life cycle, highlighting the importance of understanding how startups use social media at different stages of their development.

## Introduction

Social media platforms enable the creation of communities, provide easy access, and help companies promote their business. Their usage implies only a small investment, driving startup companies to use it as a cost-effective tool to create a digital gateway for finding customers and raising funds. The last are two of the three critical startup challenges reported by Wang et al. (2016). Building the product is the third. These challenges derive from a startup company’s fast pace of growth, making it difficult to identify the correct steps to take for scaling up. Gulati and DeSantola (2016) explain that startups can improve their growth and achieve their objectives by understanding the best scaling practices.

The definition of what is a startup company has evolved over time. The definition introduced by Lugović and Ahmed (2015a) involves two perspectives: one concerning the business dimension and the other concerning the company’s characteristics. Regarding the business dimension, if a company has been established for less than one year and employs at least one person besides its founders, then it can be considered a startup. As for the company characteristics, it must be an innovative and growth-oriented business. However, more recent work suggests that the startup definition depends on the actual stage of the company’s life cycle (Skala 2019). Therefore, the startup definition is not entirely settled, but some perspectives enable the characterization of these small companies.

Undoubtedly, social media has become a fundamental part of the information ecosystem, generating a large amount of data. Social media data can provide information about clients, products, and the overall market, improving the decision-making processes. However, data have to be processed, structured, and interpreted to infer relevant decision information. Understanding social media can help improve the company’s investment (ROI) while enabling better customer relationship management (CRM), which is supported by recent studies that focus on social media data, considering it a strategic knowledge source for businesses (Kapoor et al. 2018). Previous studies explored digital platforms startups’ data to extract relevant information about their activity. Saura et al. (2019) examined tweets using “#startup” to detect indicators for success and discovered the sentiment of the most common topics of tweets about startups. A broad study by Ruggieri et al. (2018) focused on finding patterns in successful startups based on their digital platforms’ presence. The authors stated that newly born startups use digital platforms because it is cost-effective.

Nonetheless, startups’ presence on digital platforms is continued since it enables the creation of a community between users and providers, which affects the scalability of the business, and opens new sources for creating value. Regarding the actual startups’ activity, Alotaibi et al. (2020) designed a framework to evaluate Twitter activity using an Arabic startup as a case study. Recent systematic literature reviews have highlighted the need for deeper research in social media intelligence (Olanrewaju et al. 2020; Smolak Lozano and Almansa-Martínez 2021). Olanrewaju et al. (2020) proposed a set of future work themes, among which we can find the need to consider the evolution state of the company and, in consequence, its life cycle stages. We aim to fill that gap and understand how a startup’s social media content changes through the different phases of its life. In other words, understand the diachronic profile that emerges from the startup’s historical social media data and analyze whether it reflects its scaling evolution.

Since Twitter is an ideal platform for small businesses like startups and where they are now massively present, we have chosen this platform as our primary research data source.^1^ In fact, even think tanks, an usual birthplace for startups (Feld and Hathaway 2020), use social media, like Twitter, to disseminate their activities and achieve funding (Castillo-Esparcia et al. 2020). Twitter differs from other social media platforms because it gives access to a global audience where users openly communicate with other users. Above all, it offers an opportunity for businesses to interact and receive instant feedback instead of acting solely as a marketing tool (Curran et al. 2011). Campos-Domínguez (2017) classifies Twitter activity as spontaneous and instantaneous, which can encourage a fluid exchange of ideas. Thus, Twitter can be looked at as a social media tool to help a business establish a network between customers, owners, and investors—providing an environment where professional content coexists with user-generated content, that is, nonexpert content (Casero-Ripollés 2018). Twitter activity is composed of tweets, which are essentially short text messages that may include images, emoticons, URLs, mentions, and hashtags. These characteristics make tweet categorization a challenging task. The textual analysis of startups’ tweets was performed using a topic modeling approach. We begin by assigning a category to a tweet by uncovering the tweet’s main topics and then studying the evolution of the tweets’ content over the startups’ life cycle. The present research differs from the existing literature by linking the results of the text analysis with each company’s life cycle stage to understand if and how the startups’ social media activity alters with its rise, maturity, and consequent change of goals.

This study focuses on the particular case of information technology (IT) startups founded by Portuguese executives or headquartered in Portugal as an illustrative case study. The rationale links with the fact that Portugal has created a distinctive ecosystem for IT startups over the latest years, mainly due to the Portuguese high-quality engineering talents and above-average English language fluency levels.^2^ Additionally, the Portuguese government has seriously engaged in innovation policies, promoting initiatives like Startup Portugal, 200 M, and business incubators, which have fostered the creation of several startups. Since 2016, investment in Lisbon-based startups has grown 30% yearly^3^ due to several successful startups and unicorns formed in Portugal. We selected eight IT startups from the Sifted 2020 Portugal startups list^4^ for this work. The chosen companies are currently at different stages in their life cycle and are considered active on Twitter. The content posted by the eight startups spans five years of analysis, from 2015 to 2020, resulting in a total of 15 577 tweets.

The remainder of the paper is organized as follows: After presenting the related work, the methodology section describes our dataset, presents the methodology, and proposes a new model for the life cycle of a startup business. After presenting and discussing the results of our analysis in the results section, implications are drawn. Lastly, the conclusions section describes the main conclusions and lays the path for future work.

## Related work

Social media platforms are essential digital marketing tools for small businesses, with half of the world’s population currently using these platforms (Castillero-Ostio et al. 2021). Saravanakumar and Suganthalakshmi (2012) denote social media marketing (SMM) as a marketing tactic that efficiently promotes brands through social media platforms. However, how can we analyze social media content and extract relevant information that demonstrates this value? This section aims to answer this question by explaining the social media analysis process and its methods and results. Additionally, we describe the startups’ life cycle since this constitutes the central hypothesis driving our research: the cycle of the startup’s life and evolution influences its social media activity.

### The social media analysis process and methods

Social media data has become a fundamental part of the data ecosystem and is a strategic knowledge source for decision-making (Kapoor et al. 2018). Some paradigmatic examples can be found in extant literature. Campos-Domínguez (2017) analyzed the research on political communication on Twitter, and Godoy-Martín (2022) investigated the use of social media by communications agencies. Nevertheless, to infer relevant information from data, one needs to prepare and process it (Dutot and Mosconi 2016). Social media intelligence (SMI) collects and analyzes relevant data to provide data-driven support for strategic decisions. SMI works as a cycle because social media constantly changes, with new users creating new content and generating more data for analysis. The main focus of SMI applications is product/service review analysis (Kapoor et al. 2018). The knowledge obtained by SMI is meant to describe the present state of social media. This focus means that if the objective is to predict outcomes and suggest future directions, a social media analytics (SMA) approach is deemed necessary (Choi et al. 2020). SMA and SMI present similar phases (Zeng et al. 2010), but the SMA methodology and results focus on the future, while SMI concerns the present.

Social media content is mainly text, and the goal of its analysis is to find relationships among data in textual documents and extract patterns to understand the themes being addressed (Jelodar et al. 2017). This goal can be achieved by analyzing the text’s sentiment or identifying the main topics. A topic is a list of words defined statistically to categorize the meaning of the text, and this process is termed topic modeling. Using topic modeling, researchers in the literature address problems in the most varied fields. There are several methods to conduct topic modeling. Among the most employed ones are latent Dirichlet allocation (LDA) by Blei et al. (2002), latent semantic analysis (LSA) by Landauer et al. (2007), and non-negative matrix factorization (NMF) by Lee and Seung (2001), both based on linear algebra, namely diverse forms of matrix factorization.

LDA is one of the most popular and widespread methods for identifying latent topics in a text (Blei et al. 2002). It identifies the (relevant) topics by using generative probabilistic models. One of the areas where it is applied is in social media topic analysis, as observed in the works of Saura et al. (2019); Yang and Zhang (2018); and Yu et al. (2019). While these studies focus on different problems, each uses topic modeling as a tool for SMA. D. Yu et al. (2019) developed a novel hierarchical topic modeling technique and mined the dimension hierarchy of tweets’ topics over tweets of different countries. Saura et al. (2019) analyzed tweets with the hashtag startup (“#startup”) and its comments. The objective was to understand the topics in those tweets and the associated sentiments. Yang and Zhang (2018) performed a similar analysis, where the authors combined topic modeling and sentiment analysis to mine the tweet’s text. They concluded that the LDA algorithm makes it easy to analyze an extensive set of tweets and obtain meaningful topics. Some other studies use topic modeling to explore and understand specific subjects on Twitter, like in the case of Barry et al. (2018), which analyzes alcoholic drinks advertising or a recent study to understand how politicians tweet about climate change by Chao et al. (2021). More recent works use topic modeling methods to examine Twitter information about COVID-19. For instance, Sha et al. (2020c) analyzed governmental and politicians’ tweets about the pandemic situation and inferred a set of topics that describe Twitter activity in the countries under analysis. Kaila and Prasad (2020) and Doogan et al. (2020) focused on tweets bearing hashtags related to COVID-19 to understand what non-government users tweet concerning the coronavirus pandemic and its global perception. While the former studies ascertain LDA as having achieved good results in analyzing Twitter posts, they also raise limitations about using the LDA algorithm with Twitter data. The two most common limitations are the tweets’ short text format and the need for preprocessing phase. Transforming a tweet into a document to perform a topic model might not be adequate because it has few words to extract topics. Therefore, most studies solve these limitations by aggregating the tweets into sets, where each collection corresponds to a document (Curiskis et al. 2020). However, some advances appear to avoid aggregation, as Xiong et al. (2018) demonstrated, where the authors proposed a short-text topic model algorithm.

### Startups and social media

Social media platforms have a global reach, are easy to access, and are low cost, enabling startups to use social media as a digital marketing gateway and observe the market. A few studies investigate the potential relationships between startups and social media platforms. Lugović and Ahmed (2015a) found a positive correlation between startups’ Twitter usage and the total investment in the source country. As previously stated, Saura et al. (2019) collected tweets presenting the hashtag startup (“#startup”). The authors aimed to relate the polarity of the tweet with the topics found within the diverse sentiments. The authors classified the tweet’s text and comments into positive, negative, and neutral. Then, the authors performed topic modeling for each polarity and found the related topics, enabling them to understand the Twitter audience sentiment of startup-related content.

Ruggieri et al. (2018) aimed to find patterns in successful innovative startups based on their digital platforms’ activity. Their study demonstrates that startups are present on digital platforms mainly because these platforms have a cost-effective performance. The authors also conclude that a community of users/providers of services is essential for the business. Such a community is fundamental for a positive impact on digital platforms, primarily on social networking websites, since that community provides positive or negative opinions about products and companies. Word-of-mouth is the everyday oral communication that creates an impression and idea about a specific subject (Keller 2007), and online opinions are called electronic word-of-mouth (eWOM), as explained by Hennig-Thurau et al. (2004). Social media platforms are ideal tools for eWOM. Chu and Kim (2011) describe that eWOM enables the creation of a large community, which allows for increased digital engagement with social interactions, such as comments, likes, and shares. The last two represent non-verbal activities, and when their quantities are large, they might help raise a positive feeling in the social media profile in question (Wolny and Mueller 2013). Additionally, social media activities can be used to understand the online organization’s reputation (Azinhaes et al. 2021a).

### The startups’ life cycle and its stages

Startups are primarily defined as fast-grow innovative businesses. According to Wang et al. (2016), the maturity evolution of a startup goes through two main stages: the learning and the growing stages. The learning stage consists of selecting a problem to solve and defining and evaluating the solution. The problem represents a real issue or obstacle for a specific target, which is solved by providing a product or service: the solution. The product concept is developed in the growing stage, followed by an implementation start leading to a working prototype. In case the prototype is successful, the startup obtains a functional product that later evolves into a mature product. However, Wang et al. (2016) emphasize that this is not a constant cycle, saying that a startup has to go through “multiple measure-learn loops.” The loops mean evaluating each step as being in the stages previously referred. Concerning startups whose main product/service is software, Nguyen-Duc et al. (2015) created a conceptual model named the hunter-gatherer, that in fact, consists of two development cycles: the “hunting” cycle consists of the idea, market, and features; the “gathering” cycle features the prototype, quality, and product. The intention is that the two cycles occur at each stage, but the dimension of the cycle differs over the startup’s life cycle. In the learning stage, the hunting cycle is more significant, while in the growing stage, the gathering cycle becomes prominent. Nevertheless, the cycles occur at each stage side-by-side; when the company obtains a mature product, the focus changes to quality matters.

## Methodology

This research follows the SMI steps framework described by Choi et al. (2020) for social media-based BI research. The process consists of four phases: “Data collection,” “Data preprocessing,” “Data analysis,” and “Validation & Interpretation.” According to this framework, the initial step was the extraction of the data from Twitter. As previously mentioned, a particular set of startups’ accounts was targeted as a case study: information technology (IT) startups founded by Portuguese or headquartered in Portugal, selling products or services based on machine learning (ML) approaches, and presenting a B2B business model. Thus, our analysis centers on eight startups from the Sifted 2020 Portugal startups list are as follows: *AttentiveMobile*, *Codacy*, *DefinedCrowd*, *Feedzai*, *Prodsmart*, *Talkdesk*, *Unbabel*, and *Virtuleap*.

**Fig. 1:**
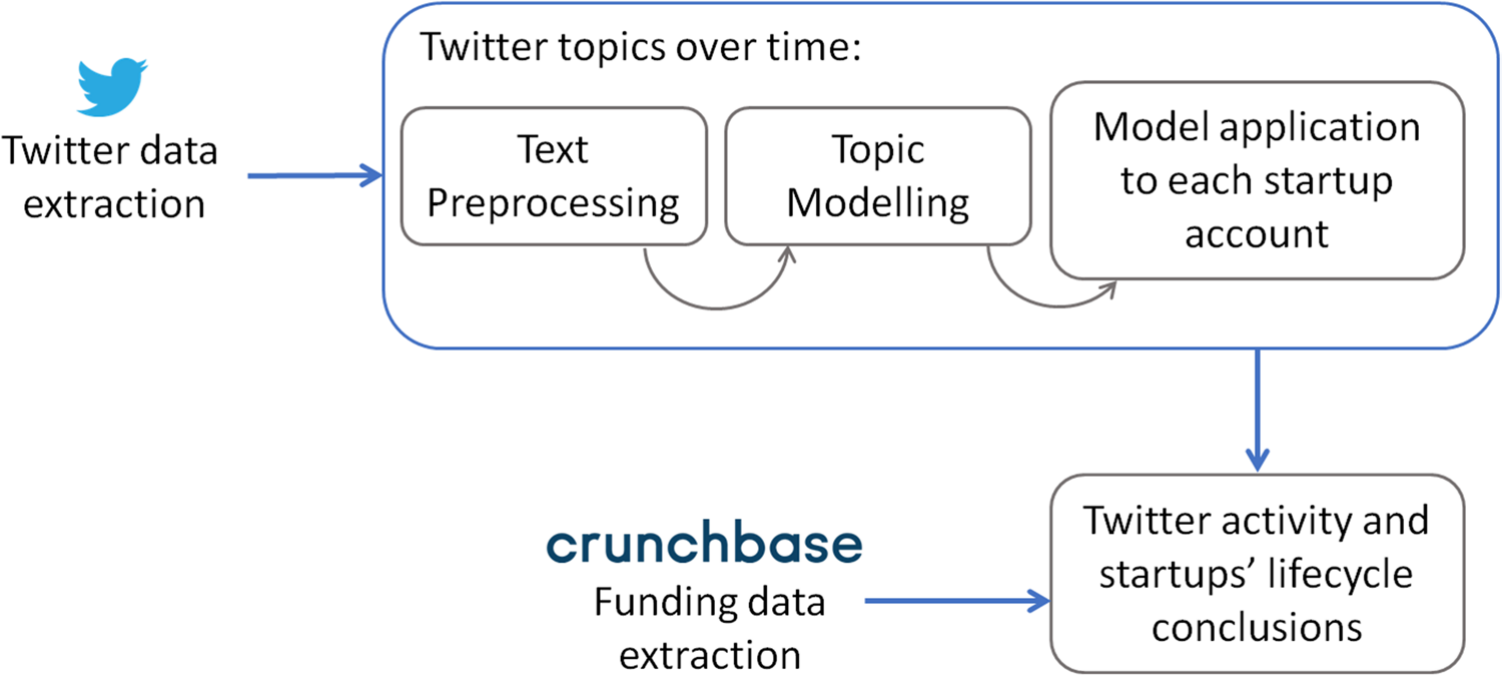
Project pipeline

After the extraction, data was cleaned, and the corpus was prepared (data preprocessing), after which we could proceed with a topic modeling (TM) technique for the analysis (data analysis). Finally, TM results are evaluated and interpreted (validation & interpretation). The latter step is where the topic modeling results are compared with the startups’ funding rounds, creating a diachronic profile for each startup. For that, the funding rounds of each startup have also been collected from Crunchbase^5^and related to the startups’ life cycle phase. Our approach is illustrated in Fig. 1.

The features that define a startup differ depending on where in the life cycle phase the company is: in the beginning, these are innovative companies with limited resources, while in the growth process, they perform an above-average rate increment in the number of customers and revenue; and finally, they have hyper-scalability and high company valuation, which characterizes a mature startup, demonstrating that startups change over their life cycle and that the definition of a startup depends on particular phases of company’s evolution (Skala 2019). Thus, a startups’ life cycle is a complex concept and, as stated by Paschen (2017), it shows two different but connected perspectives that are fundamental for the company’s success: its maturity, regarding the stage of development of a product or service, and the funding rounds, that is, the fundamental investment attraction capability.

**Fig. 2:**
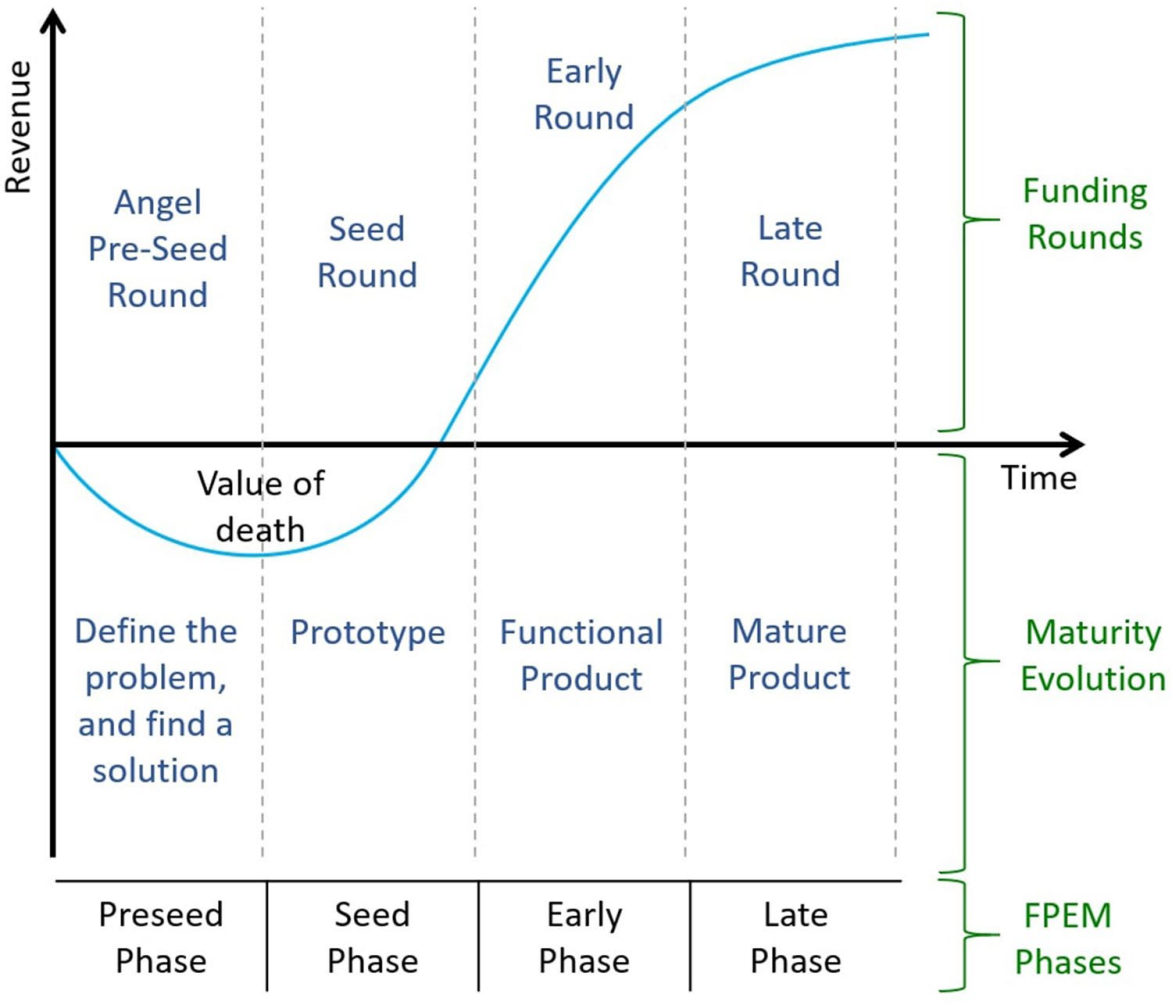
Startups’ life cycle model-funding and product evolution model (FPEM)

Based on the related literature, we consider that the startups’ life cycle can be divided into two main perspectives. One that closely follows the concepts found in Wang et al. (2016) and regards the creation of a mature product to solve a real problem: maturity evolution. Another one concerning the startup funding rounds: funding rounds. The funding rounds are where startups open or expose their shareholder structure to third parties, usually to business angels or venture capital firms, to secure investment and allow the startup to grow (Paschen 2017). To illustrate a startup’s financing milestones and evolution, we propose a life cycle model based on the previously introduced two dimensions: the funding rounds and the maturity evolution. We believe that the Funding and Product Evolution Model (FPEM), depicted in Fig. 2, illustrates the maturation process of a startup’s life regarding time and revenue in a typical success scenario.

For the model, the names of the funding rounds dimension are based on the Crunchbase Glossary,^6^ and in the maturity evolution, the phases describe the startup’s product stages based on the work of Wang et al. (2016) and Paschen (2017). The proposed model, FPEM, encompasses four key phases named after the funding round categories: the preseed phase, the seed phase, the early phase, and the late phase. For the creation of the model, we correlated the phases with the existing funding types since these are measurable, which is essential to be able to mark when a transition occurs. Then, we connected the product maturity evolution with each of the rounds. Therefore, a phase transition occurs with a funding round of a higher rank than the previous one, implying a scale-up for the company and a product maturity evolution. Typically, startups receive new funding when their product has evolved and created value for the company. However, every type of funding round can happen more than once throughout a company’s life. Notice that, for each phase, the association of concepts between maturity dimensions and funding rounds is relatively straightforward.

In the preseed phase, there is only the conceptualization of a potential and innovative solution for a concrete problem. Thus, funding is usually very limited (typically below $150 K) because it finances only an idea. These funding laps are known as angel or preseed rounds and are generally used to jump-start the company, providing financial cash to build a prototype. According to Wang et al. (2016), in this phase, the startup is in its learning stage. Next, in the seed phase, a prototype, or at least a proof-of-concept, already exists, sustaining the seed funding, which can scale up to $2 M. This round is used to build a product as market ready, incorporating the novelty proposed by the startup in the previous phase. In the early phase, the company already has a functional product and is prepared for scaling in the market. In this phase, the startup evolves for the growing stage (Wang et al. 2016). The early funding rounds, also called Series A and Series B, can have values ranging between $1 and $30 M. Lastly, in the late phase, a mature product is already established. The correspondent funding, also called Series C round, usually shows values that may start at $10 M with no upper limit.

The above-described relations between product maturity and funding rounds that represent the proposed life cycle model are validated by the topic model approach we have obtained, whose results are discussed in Sect. 4. The relations mentioned above enable us to relate each of the four FPEM phases with the uncovered topics extracted from the tweets posted by the startups on social media during their existence.

### Dataset

The dataset consists of 15 577 tweets extracted from the chosen Portuguese startups’ Twitter accounts. The date of extraction date January 10, 2021, and the data covers every tweet posted by each startup since its Twitter profile creation date. The Twitter API method was employed (“GET statuses/user_timeline”) to extract all the tweets posted by providing each company account’s username through the library tweepy (Roesslein 2020). The analysis focuses on the last five years, where the higher quantity of posts is concentrated from January 2015 to December 2020, that is, for 72 months. To accurately examine the startups’ activity over time, Table 1 shows the startup’s Twitter accounts’ descriptions.

**Table 1:** Dataset description

Company name	Founded date	First tweet date	Followers	Number of tweets	Tweets per month
Attentive mobile	2016	08/02/2018	1115	695	20.44
Codacy	2012	02/10/2013	2796	640	22.78
Defined crowd	2015	04/02/2016	1674	1258	21.69
Feedzai	2011	23/10/2015	2630	3177	51.24
Prodsmart	2012	04/12/2012	897	211	2.93
Talkdesk	2011	26/06/2019	6586	3211	178.39
Unbabel	2013	17/11/2013	3510	2615	36.32
Virtuleap	2018	29/08/2016	791	2765	53.17

It presents the company’s first tweet available date, the number of followers, the number of tweets since January 2015, and the frequency per month. The last value regards the 72 months of analysis, or the number of months since the first tweet available date if it is more recent than January 2015. Additionally, the table shows the startup founding year, collected from Crunchbase.

**Fig. 3:**
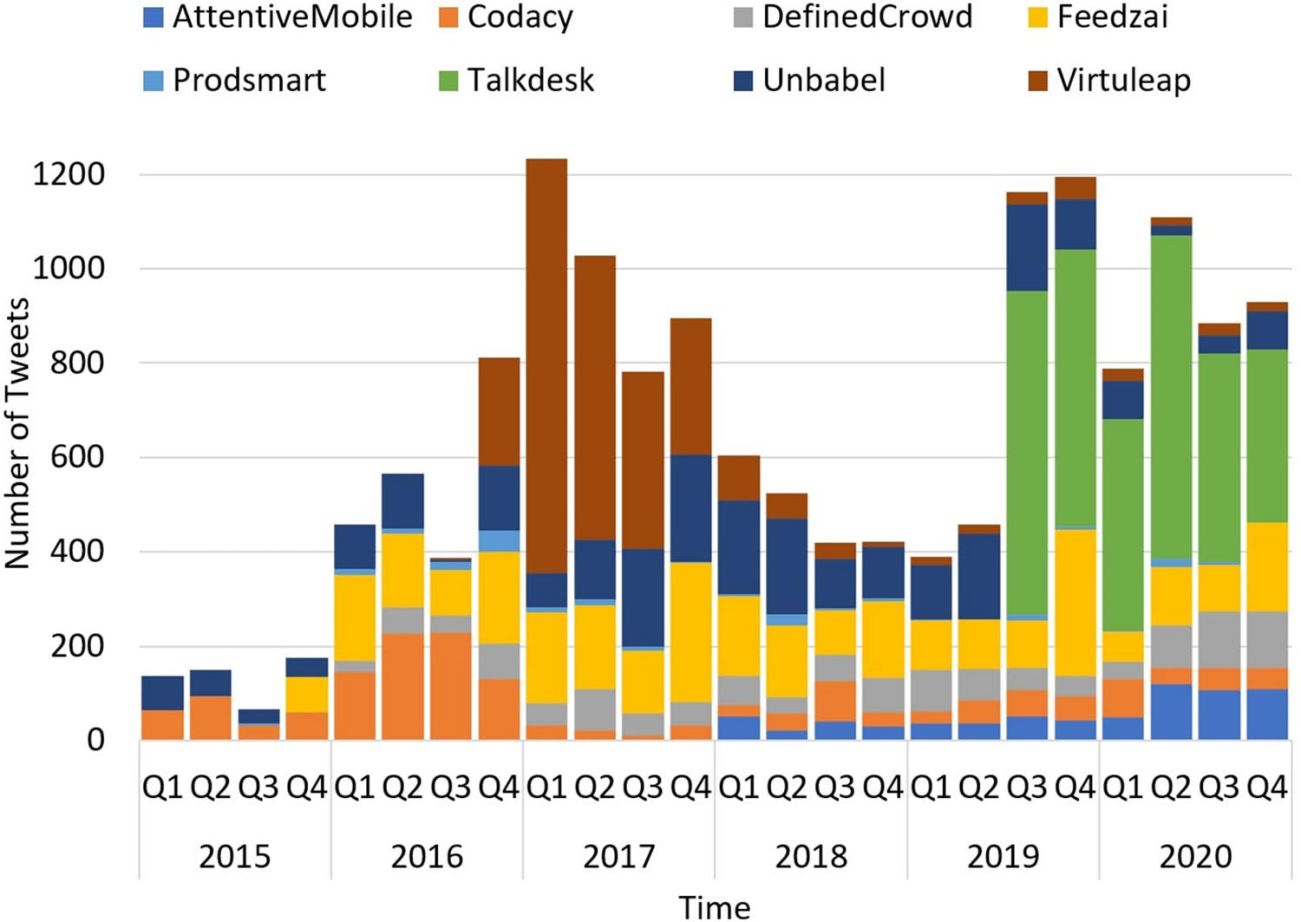
Distribution of tweets quantity over time

Figure 3 shows each startup’s quantity of tweets distributed over our chosen time window. It is possible to see that some startups post tweets regularly, while others present peaks with more activity. Within this context, regularly means the same temporal cadence, which is the case for half of the companies in the analysis: *AttentiveMobile*, *DefinedCrowd*, *Feedzai*, *Talkdesk*, and *Unbabel.* Particularly, *Talkdesk* account presents a higher number of tweets per month.

However, not every startup has presented tweet posts since the beginning of 2015. In the cases of *AttentiveMobile*, *DefinedCrowd*, *Feedzai*, and *Talkdesk*, the date for their first tweet available are more recent (Table 1 and Fig. 3). This inexistence of tweets may be because the company’s foundation date is posterior or because more ancient tweets were voluntarily deleted. Namely, *Feedzai* and *Talkdesk* are the “oldest” startups, dating from 2011, but the overall number of postings is not that high, which might suggest that they may have deleted some of their oldest tweets.

*Codacy*, *Proadsmart*, and *Virtuleap* do not post regularly, and *Virtuleap* is the only company whose activity does not cover the 72 months of the analyzed time window. *Codacy* and *Virtuleap* presented a peak in 2016 and 2017, respectively. From then on, both posts regularly but used fewer tweets per month. Notably, *Proadsmart* shows a considerably lesser degree of Twitter posting activity and is the only company that does not show posts every month.

### Text preprocessing

To understand the topics of the textual tweets, we aggregated our dataset by month, resulting in a corpus (a set of documents where each document has an id and the correspondent text) of 72 documents corresponding to each month in the time-scope of the analysis. Within each document, the id regards the month and year of the tweets. This corpus was then cleaned, retaining the vocabulary that accurately represents the startups’ content to be transformed into a document-term matrix for model training.

To ensure the adequate preprocessing of tweets, we first studied the techniques applied in literature’s similar studies, thus concluding that the literature supports the need for a preprocessing phase enabling the preparation phase for achieving coherent topics. Table 2 presents the techniques that have been applied in the existing literature.

**Table 2:** Literature preprocessing techniques usage

Preprocessing technique	Choi and Park (2019)	Alash and Al-sultany (2020)	Doogan et al. (2020)	Hidayatullah et al. (2018)	Yang and Zhang (2018)
Lowercase transformation	X		X		X
HTML tags elimination	X	X		X	X
URL elimination	X	X	X	X	X
Hashtag treatment	X	X			
Remove punctuation and digits			X	X	X
Remove stop words		X	X	X	X
Lemmatization			X		
Stemming				X	X
N-Grams		X	X		
TF-IDF			X		
Remove extra white spaces	X	X	X	X	X
Remove terms with higher frequency	X	X	X	X	X
Remove terms with less frequency	X	X	X	X	X

The most used techniques are: URL elimination, extra white spaces elimination, exclusion of the terms presenting higher or lesser frequency, HTML tags elimination, and the usage of stop words are also commonly applied.

Since white spaces, URLs, and punctuation do not present information relevant to topic’s identification, they were removed from the documents. Next, lowercase transformation and lemmatization were performed. Excluding a set of stopwords, in this case, stopwords from the Natural Language Toolkit (Bird et al. 2009) help to focus the model on the relevant words that might define the text’s meaning. For this, we added the startups’ names and Twitter tags, like “RT,” which means that it is a retweet, to the set of stopwords. The lemmatization goal is to convert every word to a common base form, providing coherence to the set of words and, consequently, to the topics. Lemmatization was done via *TextBlob* library (Loria 2020). *CountVectorizer* from the Python library *scikit-learn* (Pedregosa et al. 2011) enables vectorizing the text and having some preprocessing customization like the use of n-grams and exclusion of terms. The *n* grams used ranged from 1 to 2, uni- to bi-grams, to gather terms that may appear together, for example, the bi-gram “Machine Learning.” Then, the terms that appear less than twice were excluded to prevent possible errors and misspells. Lastly, the exclusion of terms that appear in at least 80% of the tweets. Being highly frequent terms suggests that they are meaningless in terms of topic characterization.

### Topic modeling

Due to its success in Twitter topic analysis-related literature, the topic modeling method here employed was LDA, latent Dirichlet allocation (Blei et al. 2002). The first step is to transform the corpus into a document-term matrix, where each term is either a word or a bi-gram. For that, we use the frequency of the occurrence of the term/bi-gram in the document’s text and apply the LDA algorithm on the resulting matrix using the Python library *gensim* (Rehurek et al. 2011).

**Fig. 4:**
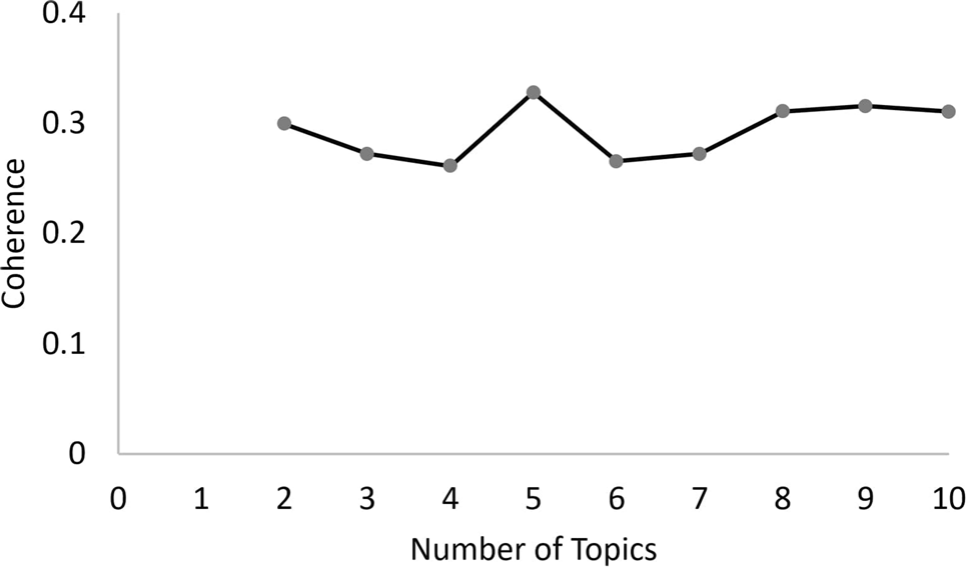
LDA coherence analysis

Since the number of topics must be given as input for the algorithm, we performed a coherence test for the advisable number of topics to be used in the modeling. Figure 4 suggests that five might be the more reliable number of topics due to its higher coherence value. Note that the coherence measure used here was *c_v*, one of the options in *gensim*.

Thus, the topic model created has five topics, each characterized by the relevant terms presented in Table 3, with all the terms showing a similar distribution within each topic.

**Table 3:** Topic description

Topic	Terms
Fintech and ML	Future, talk, fintech, banking, reality, money2020, lisbon, project, hackathon, machinelearning
Business operations	Business, cloud, opentalk2020, learn, covid19, service, solution, webinar, customer service, brand
Bank and funding	Bank, webinar, cloud, leader, learn, read, account, report, meet, partner
Product/service RD	Cloud, learn, product, read, industry, innovation, boost, service, webinar, lisbon
IT	Review, codereview, analysis, learning, websummit, machinelearning, machine learning, security, staticanalysis, lisbon

The name chosen for the first topic is “Fintech and ML” because it encapsulates “fintech’’, “machine learning,’’ and “banking,” as well as one event in this domain: “money 2020.” The second topic is “Business Operations” since it presents terms correspondent concerns typical of the company’s operations, such as “customer service,” “brand,” “solution,” and “covid19.” Additionally, it also displays “opentalk 2020,” a *Talkdesk*’s event regarding customer service subjects. “Bank and Funding” is the third topic, supported by the terms “bank,” “leader,” “report,” and “partner.” The fourth topic is “Product/Service R&D,” sustained by terms like “innovation,” “learning,” and “boost.” Lastly, “IT” (Information Technology) is the fifth topic associated with software, like code and security, and the more significant technological event, the Websummit.

## Results and discussion

After the topic model, we divided the corpus by startup and applied the model, resulting in individual analyses representing the topics’ evolution over time for each one. In order to understand if there is a relation between the FPEM phases and the Twitter activity, we combined the funding rounds’ information. The first subsection describes the results obtained per startup.

After the individual analysis, it became clear that there were similarities between the independent analysis, so we performed another study using all the startups’ data, whose results are outlined in the second subsection.

### Topics evolution over startups life cycle

The following section regards the analysis of Twitter activity over time for each company when combined with the startup’s funding rounds. Each figure shows the distribution of topics (in percentage), the number of tweets, and the funding rounds. To add context to the analysis, we provide, for each startup, a brief description of the company.

**Fig. 5:**
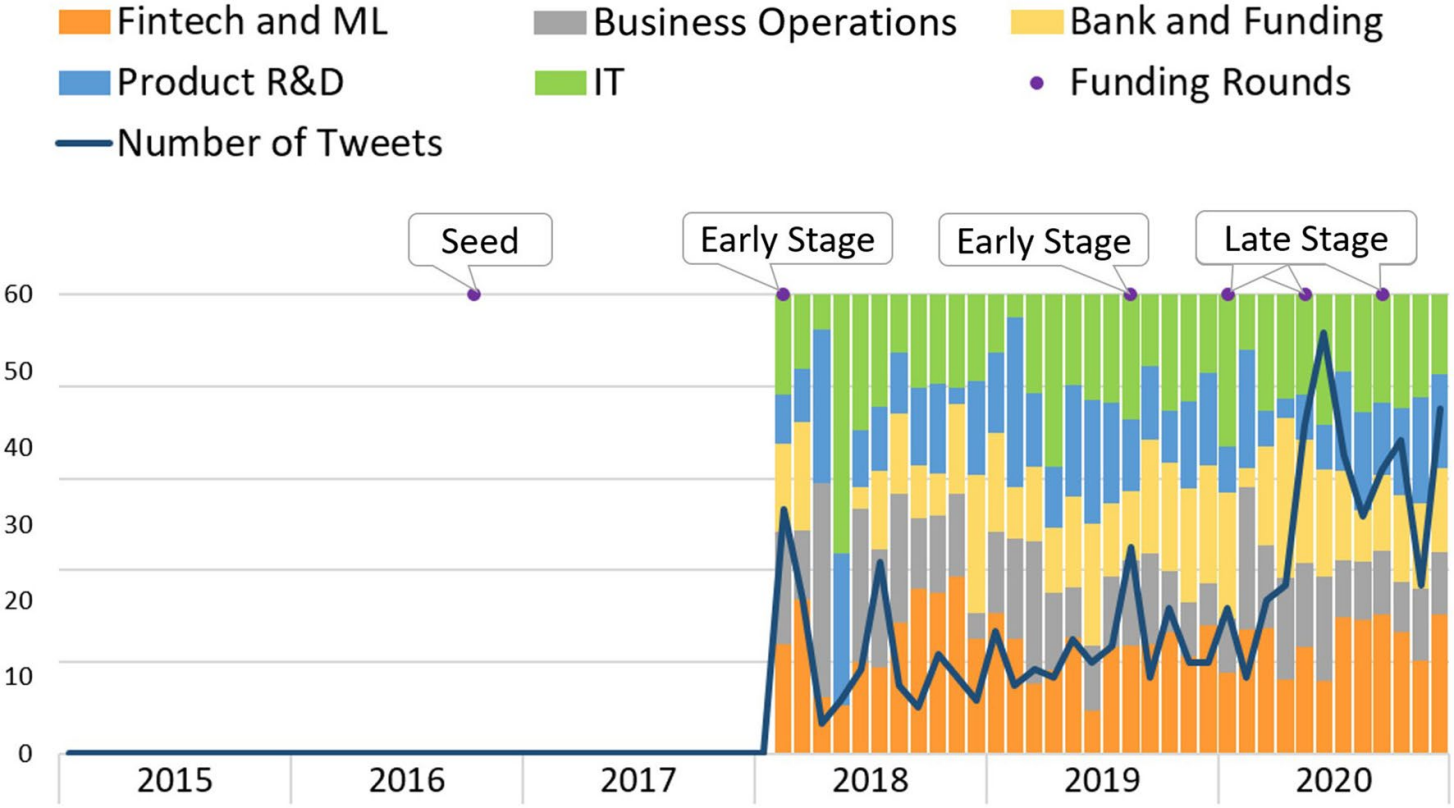
Attentive mobile

Figure 5 represents *AttentiveMobile* topics’ evolution. *AttentiveMobile* is a B2B company that offers a personalized mobile messaging platform. We can see that from 2015 until February 2018, no tweets are found. Twitter social media activity started at the startup’s early phase when the company already held a functional product. However, the topic “Product/Service R&D” is constantly present in their tweets over the years. In 2018, “Bank and Funding” was the topic less referred in their contents, but an increase can be seen over 2019, which may be because they needed new investment to grow. In fact, we can see that this topic increase precedes the company’s late stage. Nevertheless, “Fintech and ML” and “IT” topics are always present along the years and achieve half of the content posted on Twitter, clearly related to the fact the startup product is based on machine learning techniques. Since March 2020, the topics present a stationary distribution, showing a peak for the tweets’ quantity in June 2020. This scenario—higher activity numbers and stable topics’ distribution—emerges when the startup is in its late phase, where the company already owns a mature product.

**Fig. 6:**
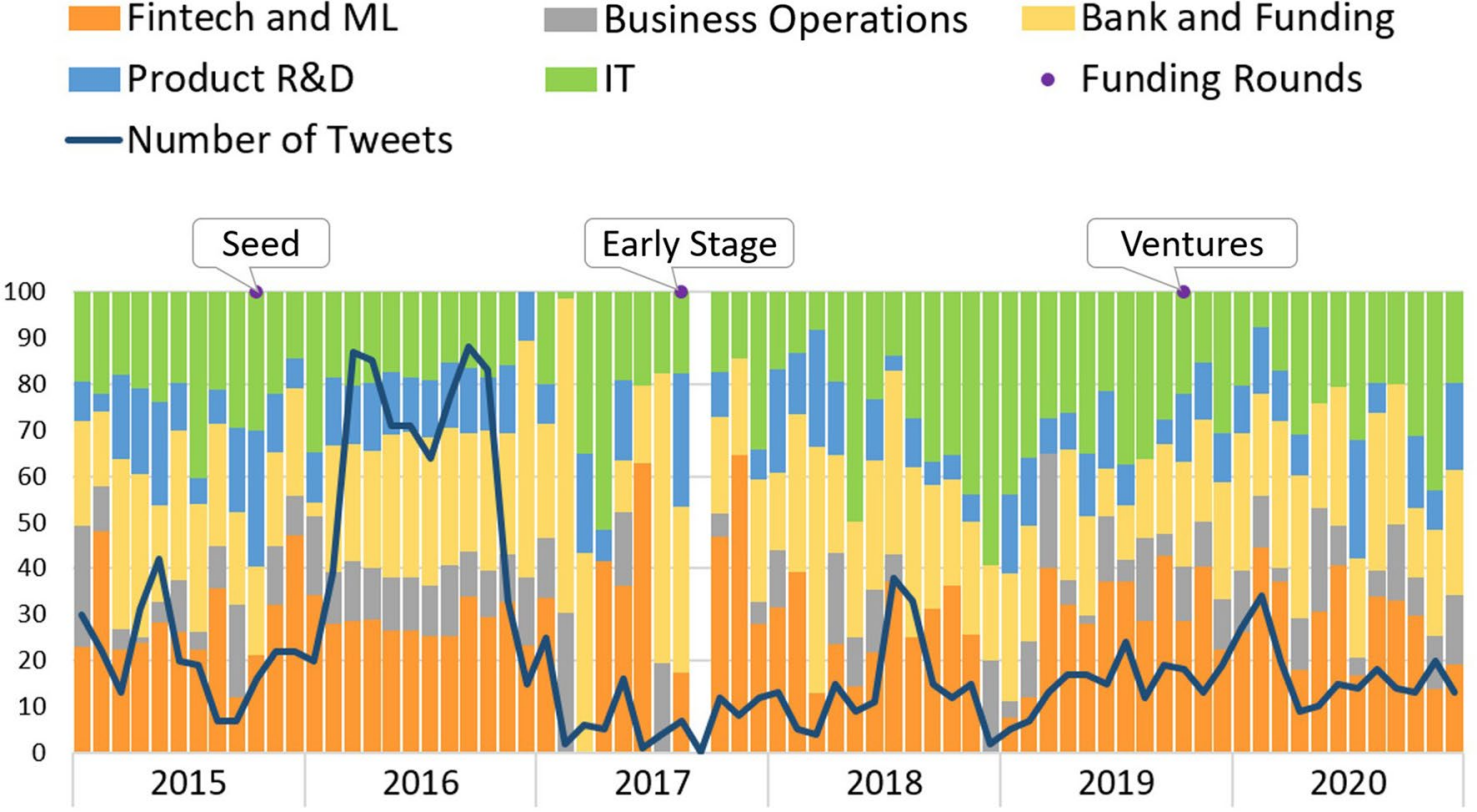
Codacy

The evolution of *Codacy* topics is depicted in Fig. 6. *Codacy* is an automated code review platform. The topics’ distribution varies over the months, but it is clear that, in 2016, the number of tweets is significantly higher showing two very similar peaks. During 2016, the startup presents itself in a seed phase, meaning it should hold a prototype. The startup changes to an early phase in August 2017, and, awkwardly, in September 2017, there were no tweets. The most predominant topics in its tweets are “IT,” “Fintech and ML,” and “Bank and Funding.” The first two may be related to the code review platform as it uses artificial intelligence methods, its core business, and the last appears associated with funding needs.

**Fig. 7:**
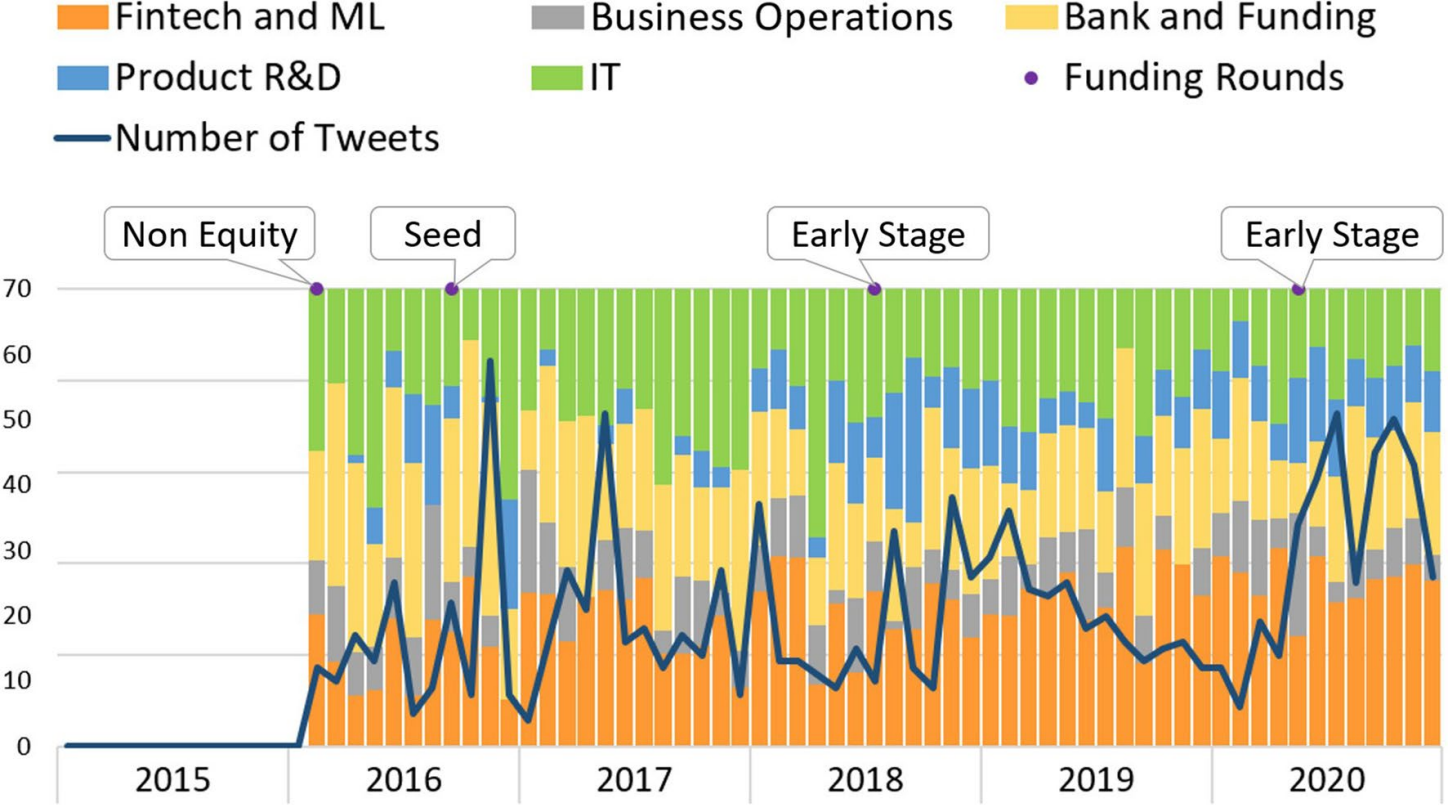
Defined crowd

*DefinedCrowd* topics’ evolution is shown in Fig. 7. This company develops artificial intelligence training data services and solutions. Although the startup’s founding year was 2015, no tweets were available from 2015 until February 2016. From then on until July 2018, when it receives the first early round, the topic distribution variability is high over those months, both in the number of tweets and for the relative representation of topics. Once it reached its early phase, the topics presented a more structured distribution, showing an increase in the “Product/Service R&D” topic in the tweets. According to the FPEM, this is a phase where, typically, companies own a fully functional product, justifying the increment in tweets related to “Product/Service R&D.” By the end of 2020, the graphic shows an increase in tweets per month, with two very similar peaks in July and in October.

**Fig. 8:**
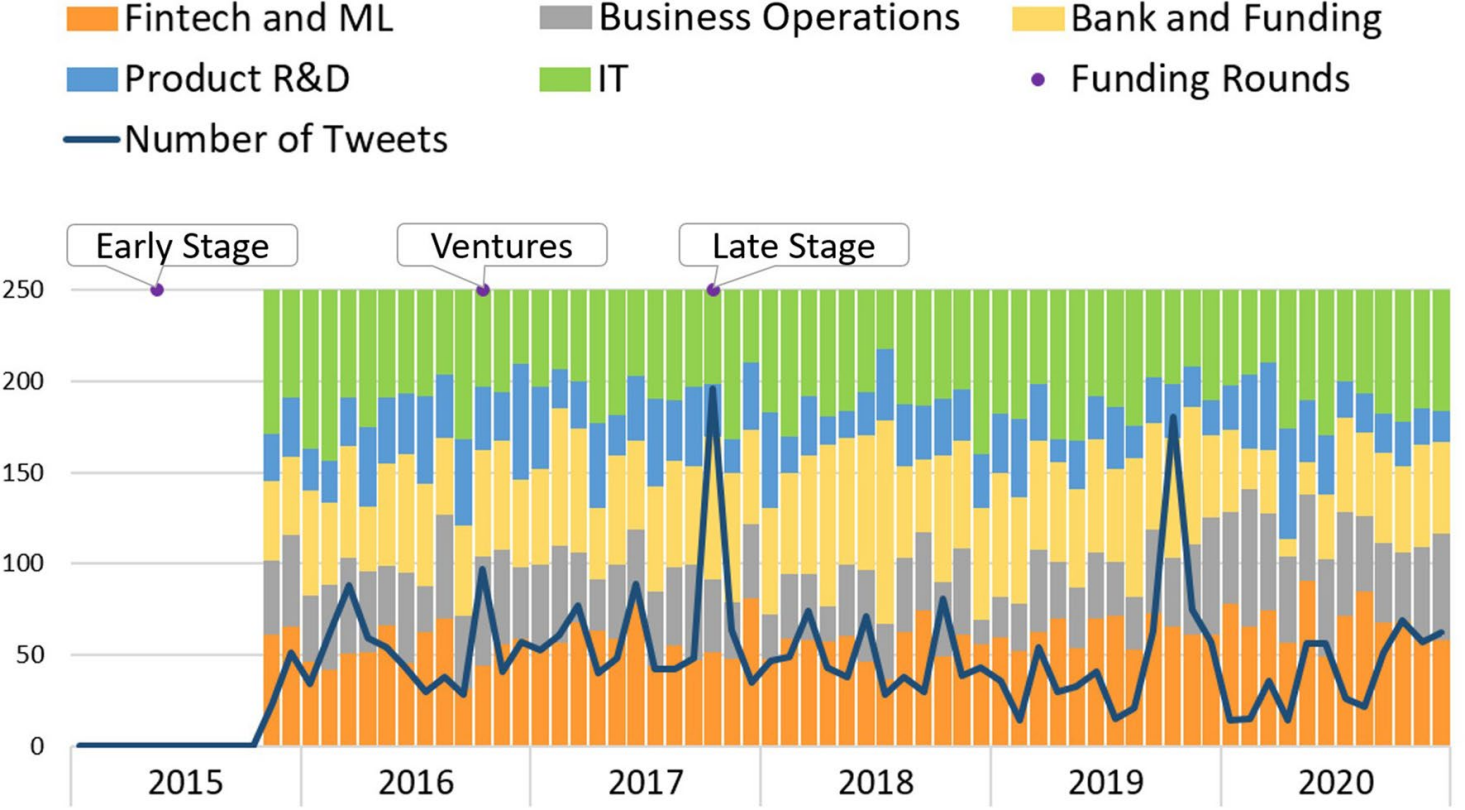
Feedzai

*Feedzai* is an artificial intelligence startup whose core business is finance risk management. *Feedzai* tweet’s profile evolution can be observed in Fig. 8. Notably, from January until November 2015, no tweets are available. From then on, Twitter’s activity starts with the company in an early phase with an already functional product. The topics show a stationary distribution, and the number of tweets is consistent over the months, except for peaks occurring in October 2017 and October 2019, possibly because of an event occurring in October. Interestingly, in 2020, the topic “Bank and Funding” shows a decrease, and “Business Operations” has increased. The decrease may be due to the fact that in October 2017, the company reached the late phase, and raising more funds was no longer a priority. Alternatively, perhaps due to the COVID-19 ongoings, the company starts posting about the pandemic instead of financial-related tweets.

**Fig. 9:**
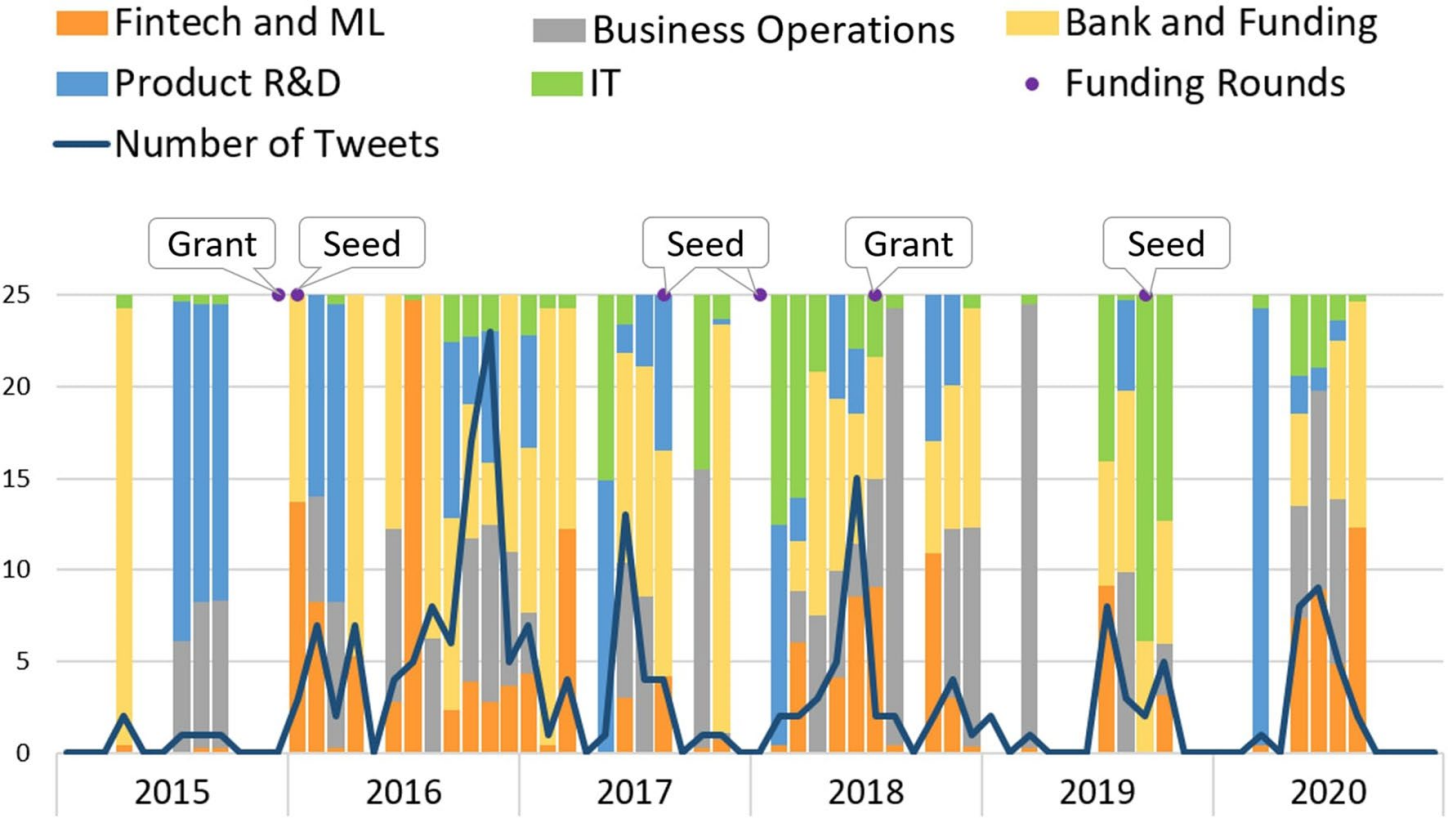
Prodsmart

*Prodsmart* turns factories into digital and smart ones by employing production automation mechanisms and controlling the workflow using their software. Figure 9 represents the company’s topics’ evolution. Not only the presence of the company in the Twitter space varies immensely, but also the tweets’ content is disparate, without any visual pattern or structure, making the distribution of the topics oscillate. During 2015, April stood out with contents relating to the topic “Bank and Funding,” while in July, August, and September of the same year, the main topics in the tweets were “Product and R&D” and “Business Operations.” Although the number of tweets is constantly lower compared with the other startups in the analysis, a peak occurred in November 2016. Since 2016, when the startup achieved the seed phase, the topics “Fintech and ML” and “IT,” representing the technology subject, started to be present in their tweets’ content. Over the years, the topic “Bank and Funding” shows a constant presence, which can be explained by the company’s funding needs since *Prodsmart* did not leave the seed phase throughout the period under analysis.

**Fig. 10:**
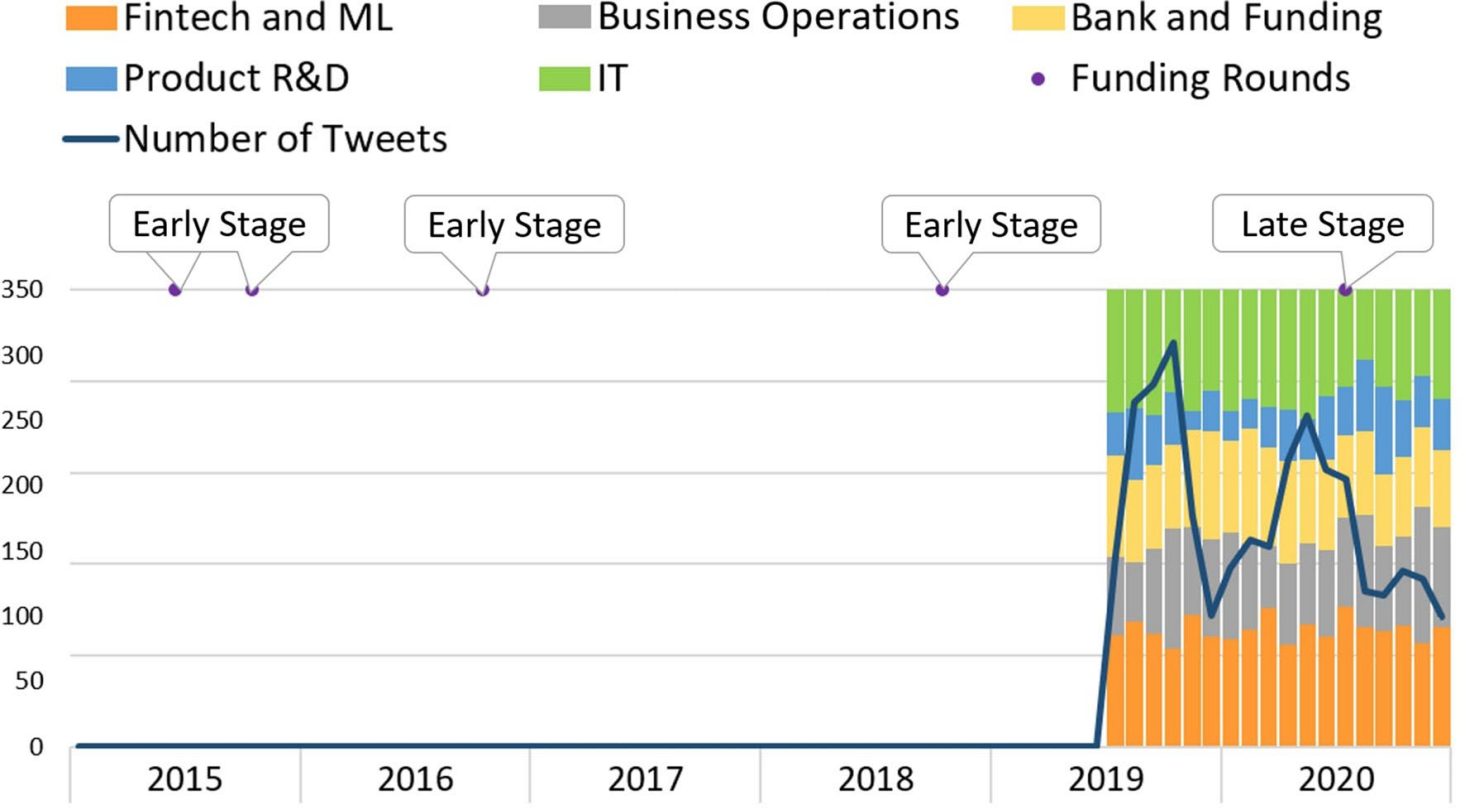
Talkdesk

Figure 10 represents the *Talkdesk* topics’ evolution. *Talkdesk* is a platform to support sales teams for costumers’ satisfaction and cost savings. Although founded in 2011, from 2015 until July 2019, no tweets were available. However, from July 2019 forward, the number of tweets is mostly above 100/month, which suggests that the company must have decided to delete previous posts. From then on, *Talkdesk* has been at an early phase, having reached the late phase in July 2020. Regarding Twitter’s activity, the topics are distributed very similarly over the months, with “Product R&D” showing the lesser number of tweets. The number of tweets shows two peaks, one in October 2019 and the other in April 2020. Since these tweets precede *Talkdesk’s* entrance into a more mature phase already involving a stable product, tweeting about product development may not be between its higher priorities.

**Fig. 11:**
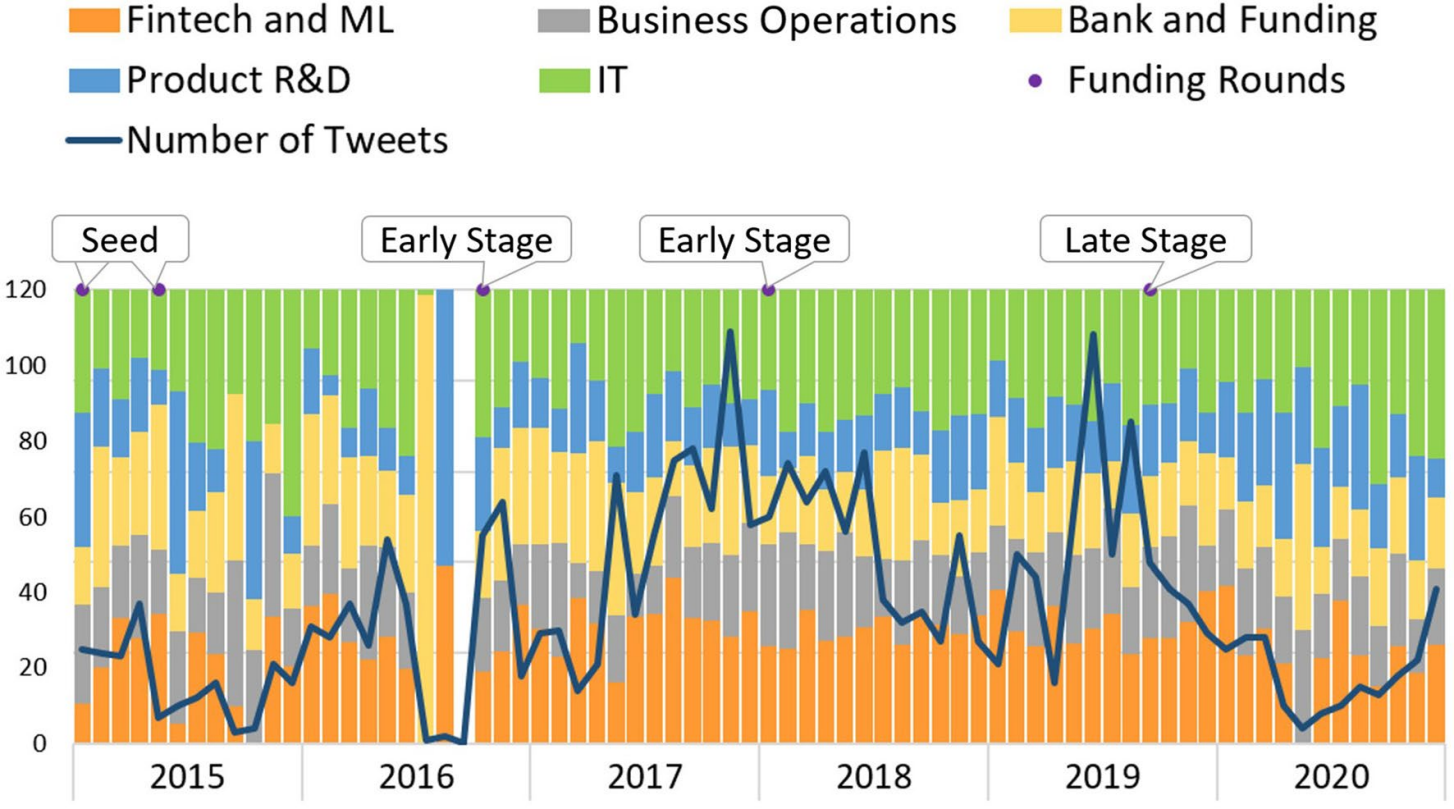
Unbabel

*Unbabel* product enables companies to serve customers in their native language with a scalable translation across digital channels. Figure 11 represents *Unbabel* topics’ evolution. The company’s first seed round was in March 2014. *Unbabel* was in a seed phase until October 2016, when it reached an early phase, followed by the late phase in September 2019. In the seed phase, the tweets’ topics show an oscillatory behavior, without a defined structure over the months, except for “IT” topic, which may be due to the heavy technological architecture of the company’s services/products. However, since the early phase, the distribution has become stationary. In September 2016, the startup showed no posts; from September 2019 until May 2020, the number of tweets has been steadily decreasing. Maybe because of the late phase the company entered, not needing to heavily promote the new product or in need of raising extra funding.

**Fig. 12:**
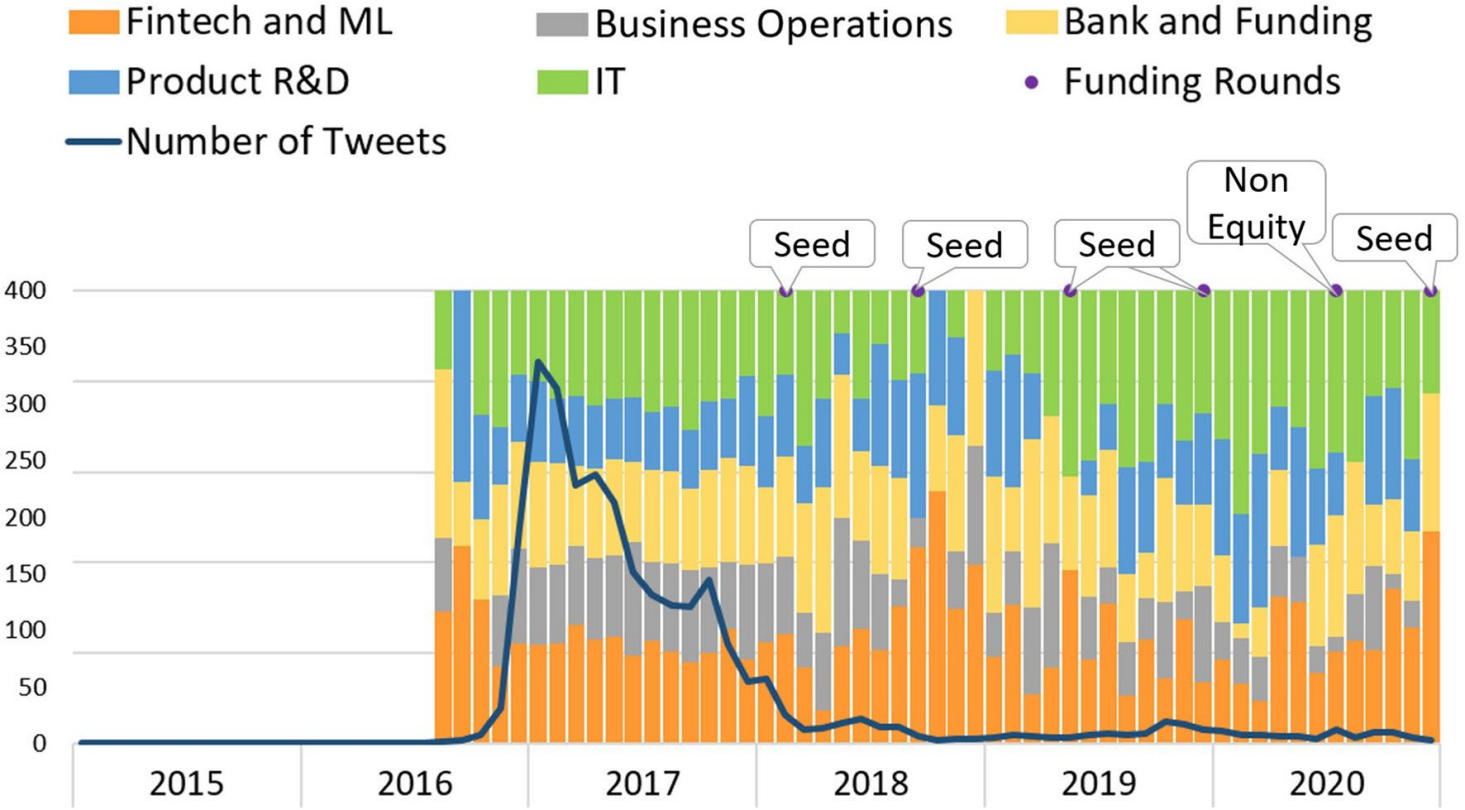
Virtuleap

Lastly, Fig. 12 depicts *Virtuleap* topics’ evolution. This company sells a virtual reality application that promotes brain health with a library of games designed by neuroscientists. From 2015 to August 2016, there were no tweets available, and it is known that the company registry occurred in 2018 with *Virtuleap* achieving a seed round in February 2018. In fact, between 2018 and 2020, the company received five seed rounds. Tweets before 2018 can be found and a high-value peak quantity of tweets occurred in January 2017, prior to the first seed round. Additionally, the topic distribution in 2017 is mostly stationary, with the topics “Fintech and ML” and “IT” having higher representation. Since 2018, the number of posts has decreased until reaching residual values by the last quarter of 2018. Regarding the topics, by the end of 2018, the tweet content starts to show higher diversity and less structure, and the topics “Product R&D” and “Business operations” decrease when compared to the previous years.

As expected, being all of these classified as IT startups, all the companies show a good percentage of the tweet’s contents addressing “IT” and “Fintech and ML.” Also prevalent throughout most of the life cycle is the “Bank and Funding” theme. Thus, next section offers a more detailed analysis of the distribution of contents in terms of the phases of the FPEM.

### Analysis of twitter activity in life cycle phases

**Fig. 13:**
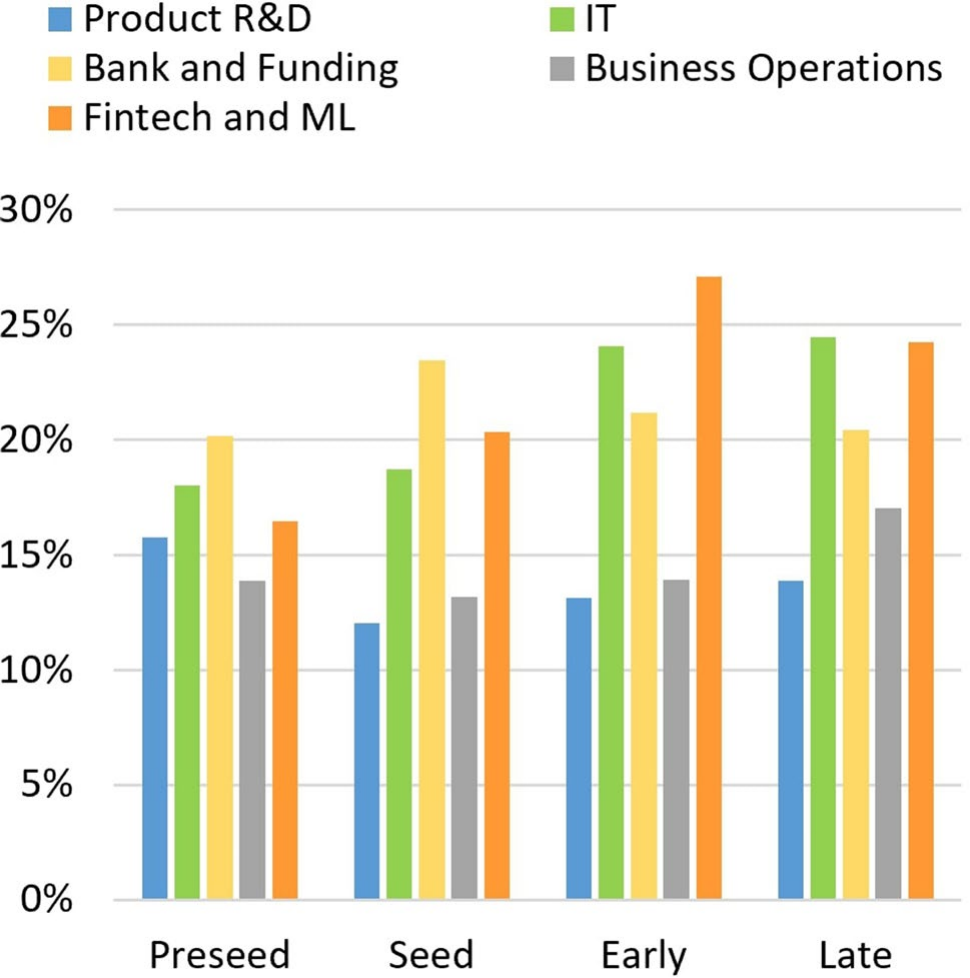
Average of the topics’ predominance per phase

The previous observations suggest that the content and the number of tweets posted by the startups may differ over their FPEM life cycle phases. It is possible to see (Fig. 13) that in terms of life cycle phases, the percentage of topics differs.

**Fig. 14:**
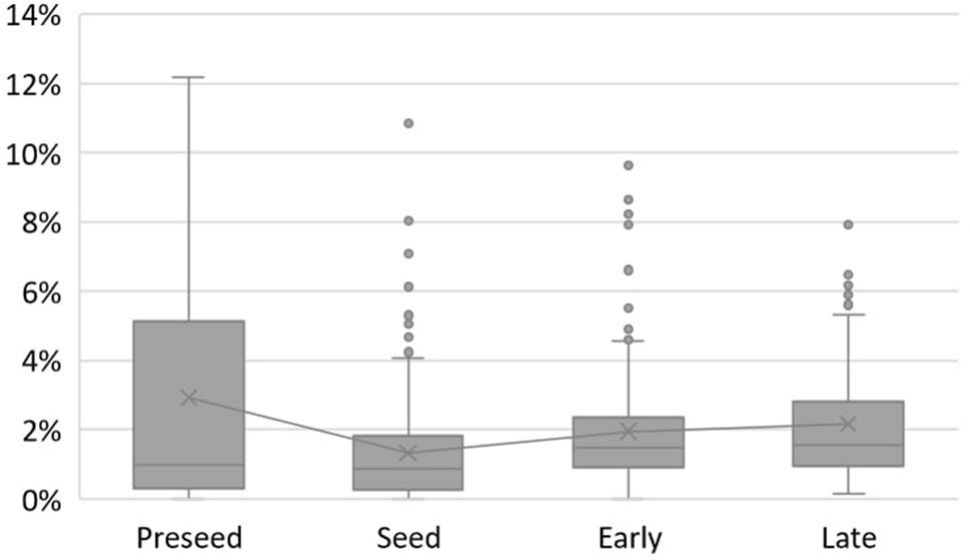
Tweets quantity over life cycle phases

As it can be seen, the topic “Product R&D” is slightly higher in the preseed phase, and “Business Operations” is more eminent in the late phase. Newer companies need to focus on product development and in its promotion, while more mature startups already hold a final product in the market, allowing them to prioritize business concerns. The topics “Fintech and ML” and “IT” have similar distribution over all the life cycle phases, although showing a higher percentage in early and late phases. Lastly, the topic “Bank and Funding” shows to be the more constant theme, averaging about 20% for all posts. Concerning newer companies, in preseed and seed, those post more about the topic “Bank and Funding,” demonstrating the importance that financing has for their growth. In contrast, companies in early and late phases post more about the technology applied in their product, corresponding to the topics “Fintech and ML” and “IT.” Additionally, the preseed phase is the one with minor variance between the topics’ percentages over the phases, showing that for companies that at in this stage of their life cycle may have a specific focus for their Twitter content, since they tend to post more (Fig. 14) and more consistently.

To understand if the relative emergence of topics within tweets differs according to each of the four FPEM phases, and since we have no good reason to assume that the topics distribution follows a normal distribution, the Kruskal–Wallis test was used. This is a nonparametric method that compares the means between groups, which in this scenario will be the four life cycle phases. We used the *SciPy* (Virtanen et al. 2020) library for implementing the Kruskal–Wallis test, setting the significance threshold at 0.05. The null hypothesis states that the means in each life cycle phase are the same. If the *p-value* is lower than the threshold, we reject the null hypothesis, meaning that the means on every life cycle are not the same. The results are presented in Table 4, denoting the ones with a *p*-value below the significance with (*).

**Table 4:** Kruskal–Wallis tests results

	*p*-value
Topic: product R&D	(*)$0.00545$
Topic: IT	(*)$2.38\mathrm{E}-13$
Topic: bank and funding	$0.327$
Topic: business operations	(*)$2.4E-06$
Topic: fintech and ML	(*)$8.82E-08$
Number of tweets	(*)$2.72E-08$

(*) Statistically significant ($$p< 0.05$$)

The topics “Product R&D,” “IT,” “Business Operations,” and “Fintech and ML” present a *p-value* lower than the threshold, meaning that their means differ over the life cycle phases. “Bank and Funding” is the exception on the Kruskal–Wallis test, presenting a *p-value* expressively higher than the significance. This might imply that it remains more stable over the life cycle, which is consistent with the analysis of the information depicted in Fig. 13.

The results also prove a statistically significant relationship between the number of tweets and the startup phases. This relationship can be visualized in Fig. 14, which shows the proportion of tweets posted per month and distributed into the life cycle phases. The graph shows that all the startups have posted more on average when traversing the preseed phase.

Additionally, the higher variation in the preseed phase may be due to the fact that some of the startups in the analysis have been in this phase through a big part of the data time window. However, posts from some other startups at a preseed phase were not available (or not included in the case where it occurred before 2015). Notoriously, once a seed phase is achieved, startups’ number of posts is notably less. This decrease in posting may be because they have received a funding round and are now more focused on product development. Nevertheless, the number of tweets slightly increases through the early and late phases.

**Fig. 15:**
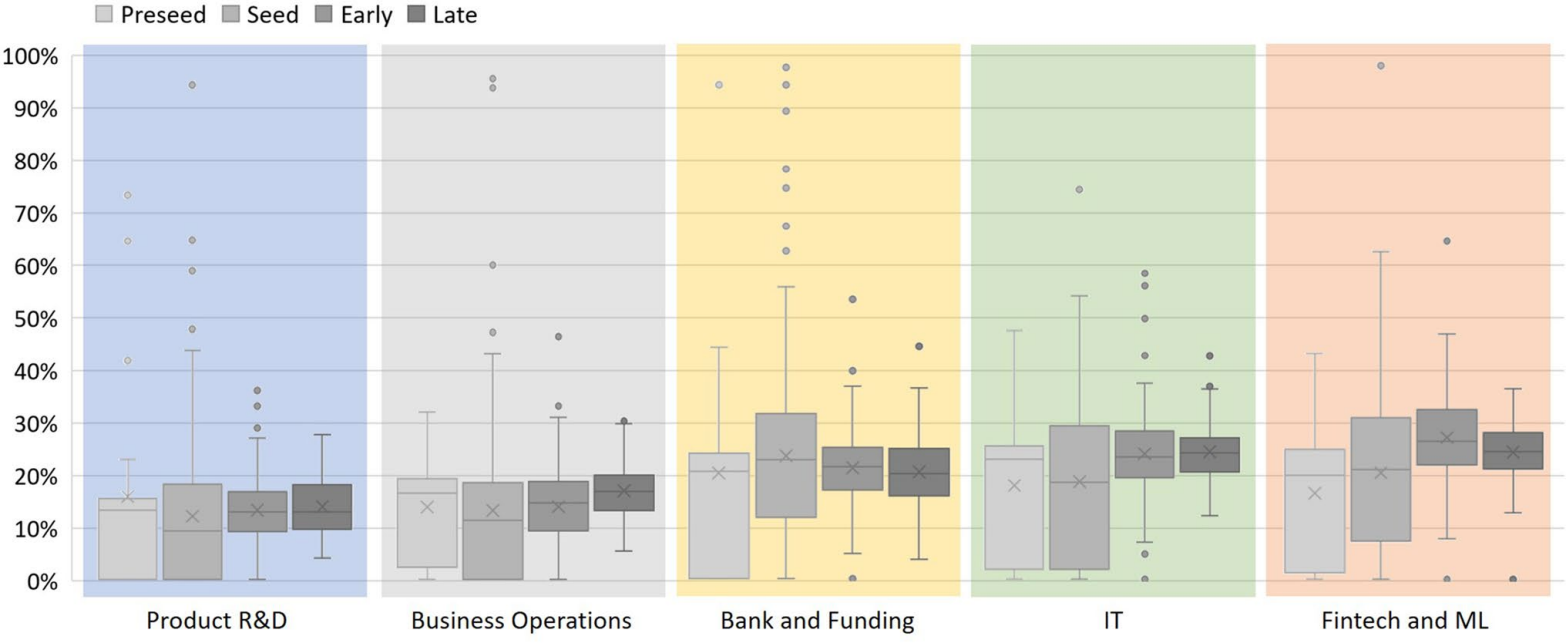
Topics distribution over life cycle phases

Figure 15 displays the distributions of each topic to understand how, accordingly to the Kruskal–Wallis test, they differ throughout the FPEM phases. The topic “Product R&D,” with the rejection of the null hypothesis, means that the distribution varies through the life cycle phases. In fact, this topic presents means higher values for the preseed phase and lower ones in the subsequent ones. This change can illustrate the importance of product development in the startups’ beginning and confirms the maturity stage correspondent state in the life cycle description of FPEM. That is, startups in the preseed phase are finding a solution to a problem. The topic “Business Operations,” which means they differ over the life cycle phases, has lower values in preseed and increases over the following phases. Having the opposite behavior of “Product R&D” and showing that with the startup growth, content about product development is exchanged by business concerns. The topics “IT” and “Fintech and ML,” related to the startups’ core business in the analysis, have a similar evolution over the phases. Both topics increase until the early phase and lightly decrease in the late phase. Note that those have a statistical significance to support the mean difference over the life cycle. Lastly, the topic “Bank and Funding” is the only means that do not differ over the phases, always staying around 20% value. The constant presence of this topic demonstrates the importance of fundraising and financial matters for startups and supports the fact that funding rounds are a dimension that characterizes startups.

## Implications

The primary goal of this study was to understand how Twitter contents of IT startups evolve over the company’s growth. Literature shows that startups experience characteristic phases due to companies changing through their life cycle and adjusting their goals.

The first contribution of this study is the conceptualization of a life cycle model. This proposal is based on two dimensions previously described in the literature: maturity evolution and funding rounds. Maturity regards the development of the product or service the startup is selling, and the funding rounds regard capitalization through investors’ financing. Our proposal unites those dimensions, creating a natural flow of business evolution: the funding and product evolution model (FPEM).

The second important implication of this study is categorizing IT startups’ social media activity. Understanding the Twitter content was achieved through topic modeling, leading to a well-defined set of five topics describing the main subjects in the startups’ tweets, which are “Fintech and ML,” “IT,” “Business Operations,” “Product/Service R&D,” and “Bank and Funding.”

The third implication brings light to the question of how the startup’s phases within its life cycle may affect social media usage. Our findings suggest that Twitter content produced by IT startups changes over the FPEM phases, while the startups scale up. The results outline that startups’ initial posts are primarily related to product development and, in more advanced maturity phases, tweets became related to operations and business concerns. As expected, one of the topics found, “Bank and Funding,” constantly emerge in tweets over the entire life cycle, denoting financial matters are a cornerstone for startups, as should be expected due to the particularities of these companies.

## Conclusions and future work

This study proposes a new startup’s life cycle model based on funding rounds and the companies’ product maturity: the Funding and Product Evolution Model–FPEM. The validity of FPEM is illustrated using an SMI cycle-based methodology to extract the main topics from eight IT startups founded by Portuguese or headquartered in Portugal. The Twitter posts were subjected to an automatic information extraction of topics to understand if the tweets’ contents change while startups are scaling up. The tweets posted between 2015 and 2020 were subjected to a topic model analysis for the IT startups chosen, adding up to 15 577 selected tweets. The results were combined with the FPEM life cycle model, creating a diachronic profile for each one of the startups. It was possible to perceive that the startups’ key topics are: “Fintech and ML” and “IT,” which regard the startups’ core business; “Business Operations” and “Product/Service R&D” about enterprise subjects and product development; and “Bank and Funding” concerning startups’ financing.

Nevertheless, results reveal that IT startups’ Twitter topics change over time according to the company’s current life cycle. The number of tweets published also varies according to the startup phase, showing that newer and more mature IT startups post more on Twitter when compared to companies in an intermediate phase. In terms of content, “Bank and Funding” is the only one of the five topics present throughout a startup’s life cycle, demonstrating the great importance of financial investments and capital enabling the company’s growth. On the other hand, another uncovered topic, “Product R&D,” is predominant during the preseed phase, showing that startups begin as product-focused companies. In contrast, the topic “Business Operations” is prevalent in the late phase, revealing that business concerns take the place of the product development content with the startup’s growth. Therefore, social media content evolves with the startups’ evolution and scaling stages.

This study has several academic and practical contributions to how startups can employ social media in their growth process. Future research can map startups’ maturity and scaling using this study’s FPEM. The proposed life cycle model can guide researchers through the distinct phases. The results obtained in this study, namely the identified topics and their distribution through the startup life cycle, can be used by startups to create better marketing strategies. Regularly posting about “Bank and Funding” throughout the different phases seems to be a feasible approach. Lastly, investors can use the model proposed in this study to monitor startup’s phases based on their social media activity and improve their investment decisions.

Like all studies, the study has limitations that should be considered in future research. Firstly, it focused only on IT startups based in Portugal. Future research should explore startups from other industries and countries to confirm whether the results are similar, regardless of the industry and region. Secondly, this study relies solely on publicly available Twitter data. Future studies should use data from other social media platforms, such as LinkedIn, to understand if posted contents vary for different platforms or if complementary topics emerge. Thirdly, the startups in this study were at different FPEM phases, which may have limited the possibility of a complete startup life cycle for some. Therefore, future research could focus on studying other startups at the same phase of the FPEM for more comprehensive results. Finally, we only validated the FPEM with the topics extracted from social media. Future work must use other data sources concerning startups to revalidate the model, like interviews with founders and venture capital experts.

## Footnotes

1 https://www.thebalancesmb.com/top-reasons-why-your-small-business-should-use-twitter-2948523.

2 https://www.ef.com/wwen/epi/.

3 https://beportugal.com/startup-in-portugal/.

4 https://sifted.eu/portugal-startups-top-rankings/.

5 www.crunchbase.com

6 https://support.crunchbase.com/hc/en-us/articles/115010458467-Glossary-of-Funding-Types.

## References

[CR1] Alash HM, Al-Sultany GA (2020). Improve topic modeling algorithms based on twitter hashtags. J Phys Conf Ser.

[CR2] Alotaibi B (2020). Startup initiative response analysis (SIRA) framework for analyzing startup initiatives on twitter. IEEE Access.

[CR3] Azinhaes J, Batista F, Ferreira JC (2021). EWOM for public institutions: application to the case of the Portuguese army. Soc Netw Anal Min.

[CR4] Barry AE, Valdez D, Padon AA, Russell AM (2018). Alcohol advertising on twitter—a topic model. Am J Health Educ.

[CR5] Bird, Steven., Ewan. Klein, and Edward. Loper. 2009. Natural language processing with Python Natural Language Processing with Python. O’Reilly. https://www.oreilly.com/library/view/natural-language-processing/9780596803346/ (January 16, 2023)

[CR6] Blei DM, Ng AY, Jordan MT (2002). Latent Dirichlet allocation. Adv Neural Inf Process Syst.

[CR7] Campos-Domínguez E (2017). Twitter y La comunicacíon política. In El Profesional De La Información.

[CR8] Casero-Ripollés A (2018). Research on political information and social media: key points and challenges for the future. El Prof De La Inform.

[CR9] Castillero-Ostio E, Gil-Ramírez M, Castillo-Esparcia A (2021). Redes Sociales Como Espacios Comunicativos de Articulación de Movimientos Sociales: Revolución de Los Frijoleros (Guatemala). Chasqui Revista Latinoamericana De Comunicación.

[CR10] Castillo-Esparcia A, Castillero-Ostio E, Castillo-Díaz A (2020). Los Think Tanks En España. Análisis de Sus Estrategias de Comunicación Digitales. Revista Latina.

[CR11] Chao Y, Margolin DB, Fownes JR, Eiseman DL, Chatrchyan AM, Allred SB (2021). Tweeting about climate: which politicians speak up and what do they speak up about?. Social Media + Society.

[CR12] Choi HJ, Park CH (2019). Emerging topic detection in twitter stream based on high utility pattern mining. Expert Syst Appl.

[CR13] Choi J (2020). Social media analytics and business intelligence research: a systematic review. Inform Proc Manag.

[CR14] Chu S-C, Kim Y (2011). Determinants of consumer engagement in electronic word-of-mouth (EWOM) in social networking sites. Int J Adv.

[CR15] Curiskis SA, Drake B, Osborn TR, Kennedy P (2020). An evaluation of document clustering and topic modelling in two online social networks: twitter and reddit. Inform Proc Manag.

[CR16] Curran K, O’Hara K, O’Brien S (2011). The role of twitter in the world of business. Int J Bus Data Commun Netw.

[CR17] Doogan C, Buntine W, Linger H, Brunt S (2020). Public perceptions and attitudes toward COVID-19 nonpharmaceutical interventions across six countries: a topic modeling analysis of twitter data. J Med Internet Res.

[CR18] Dutot V, Mosconi E (2016). Social media and business intelligence: defining and understanding social media intelligence. J Decis Syst.

[CR19] Emilia SL, Almansa-Martínez A (2021). Estudio de La Producción científica sobre social media. El caso de las revistas españolas de comunicación en JCR y SJR. Revista De Ciencias De La Comunicación e Información.

[CR20] Feld B, Hathaway I (2020). The startup community way: evolving an entrepreneurial ecosystem.

[CR50] Godoy-Martín (2022) Las agencias de comunicación ante las nuevas redes sociales. ¿Early adopters o incorporación tardía? Revista Internacional de Relaciones Públicas 12(23):225–244. 10.5783/RIRP-23-2022-12-225-244

[CR21] Gulati R, Alicia DS (2016) “Startups that last.” Harvard Business Review 2016(March). https://hbr.org/2016/03/startups-that-last (January 16, 2023)

[CR22] Hennig-Thurau T, Gwinner KP, Walsh G, Gremler DD (2004). Electronic word-of-mouth via consumer-opinion platforms: what motivates consumers to articulate themselves on the internet?. J Interact Mark.

[CR23] Hidayatullah AF et al. (2018). “twitter topic modeling on football news.” In: 2018 3rd international conference on computer and communication systems, ICCCS 2018: 94–98

[CR24] Jelodar H (2017). Latent dirichlet allocation (LDA) and topic modeling: models, applications, a survey. Multimed Tools Appl.

[CR25] Kaila RP, Prasad AVK (2020). Informational flow on twitter-corona virus outbreak–topic. Int J Adv Res Eng Technol (IJARET).

[CR26] Kapoor KK (2018). Advances in social media research: past, present and future. Inf Syst Front.

[CR27] Keller Kd (2007). Unleashing the power of word of mouth: creating brand advocacy to drive growth. J Adv Res.

[CR28] Landauer TK, McNamara DS, Dennis S, Kintsch W (2007). Handbook of latent semantic analysis.

[CR29] Lee Daniel D, Sebastian Seung H (2001) “Algorithms for non-negative matrix factorization.” advances in neural information processing systems 13. https://proceedings.neurips.cc/paper/2000/file/f9d1152547c0bde01830b7e8bd60024c-Paper.pdf (January 16, 2023)

[CR30] Loria S (2020b) “TextBlob: simplified text processing—TextBlob 0.16.0 documentation.” https://textblob.readthedocs.io/en/dev/ (January 16, 2023)

[CR31] Lugović S, Wasim A (2015a) “An analysis of twitter usage among startups in Europe.” In: 299–308. Lugovic, ahmed, an analysis of twitter usage among startups in EU.pdf. http://infoz.ffzg.hr/infuture/2015a/images/papers/8-02

[CR32] Nguyen-Duc A, Seppänen P, Abrahamsson P (2015). Hunter-gatherer cycle: a conceptual model of the evolution of software startups. ACM Int Conf Proc Ser.

[CR33] Olanrewaju AS, Temitope MA, Hossain NW, Mercieca P (2020). Social media and entrepreneurship research: a literature review. Int J Inf Manage.

[CR34] Paschen J (2017). Choose wisely: crowdfunding through the stages of the startup life cycle. Bus Horiz.

[CR35] Pedregosa F et al. (2011) 12 Journal of machine learning research scikit-learn: machine learning in python (2023).http://scikit-learn.sourceforge.net

[CR36] Rehurek R, Sojka P (2011). Gensim–python framework for vector space modelling.

[CR37] Roesslein J (2020) “Tweepy: twitter for python!” https://github.com/tweepy/tweepy (January 16, 2023)

[CR38] Ruggieri R (2018). The impact of digital platforms on business models: an empirical investigation on innovative startups. Manag Mark.

[CR39] Saravanakumar M, Suganthalakshmi T (2012). Social media marketing. Life Sci J.

[CR40] Saura JR, Palos-Sanchez P, Grilo A (2019). Detecting indicators for startup business success: sentiment analysis using text data mining. Sustainability (switzerland).

[CR41] Sha H, Hasan MA, Mohler G, Jeffrey Brantingham P (2020). “Dynamic topic modeling of the COVID-19 twitter narrative among U.S. Gov Cabinet Exec.

[CR42] Skala A (2019). Digital startups in transition economies: challenges for management, entrepreneurship and education.

[CR43] Virtanen P (2020). SciPy 1.0: fundamental algorithms for scientific computing in python. Nat Methods.

[CR44] Wang X (2016). Key challenges in software startups across life cycle stages. Lect Notes Bus Inform Proc.

[CR45] Wolny J, Mueller C (2013). Analysis of fashion consumers’ motives to engage in electronic word-of-mouth communication through social media platforms. J Mark Manag.

[CR46] Xiong S, Wang K, Ji D, Wang B (2018). A short text sentiment-topic model for product reviews. Neurocomputing.

[CR47] Yang S, Zhang H (2018). Text mining of twitter data using a latent Dirichlet allocation topic model and sentiment analysis. Int J Comput Inform Eng.

[CR48] Yu D, Dengwei Xu, Wang D, Ni Z (2019). Hierarchical topic modeling of twitter data for online analytical processing. IEEE Access.

[CR49] Zeng D, Chen H, Lusch R, Li SH (2010). Social media analytics and intelligence. IEEE Intell Syst.

